# Canagliflozin and Brusatol Synergize against LKB1-KEAP1 Co-Mutant NSCLC through AKT Suppression

**DOI:** 10.7150/ijbs.124757

**Published:** 2026-04-23

**Authors:** Yiguan Chen, Xiang-Zheng Gao, Dade Rong, Liangliang Gao, Mingzhu Tang, Guang Lu, Zhi-Qiang Ling, Han-Ming Shen

**Affiliations:** 1 Faculty of Health Sciences, Ministry of Education Frontier Science Centre for Precision Oncology, University of Macau, Macau SAR, China.; 2 Department of Physiological Zhongshan School of Medicine, Sun Yat-sen University, Guangzhou, Guangdong, China.; 3 Zhejiang Cancer Hospital, Hangzhou, Zhejiang 310022, China; Hangzhou Institute of Medicine (HIM), Chinese Academy of Sciences, Hangzhou, Zhejiang, China.

**Keywords:** NSCLC, LKB1, KEAP1, NRF2, Canagliflozin, Brusatol

## Abstract

Liver kinase B1 (LKB1, encoded by *STK11*) is an important tumour suppressor, with approximately 30% of non-small cell lung cancer (NSCLC) patients harbouring LKB1 mutations. Our previous work showed that LKB1-mutant NSCLC cells are sensitive to glucose starvation, suggesting that suppression of glucose metabolism may serve as a potential therapeutic strategy for NSCLC patients with LKB1 mutation. In this study, we found LKB1 mutations frequently co-occur with mutations in Kelch-like ECH-associated-protein 1 (KEAP1), another key tumour suppressor regulating the NRF2-mediated antioxidant response. To target LKB1-KEAP1 co-mutant NSCLC, we utilized Canagliflozin, an FDA-approved sodium-glucose co-transporter 2 (SGLT2) inhibitor that mimics glucose starvation via inhibiting glucose uptake, in combination with Brusatol, an inhibitor of NRF2 signalling. Our results demonstrate that the combined treatment of Canagliflozin and Brusatol exerts potent anti-tumour effects in LKB1-KEAP1 co-mutant NSCLC cells both *in vitro* and* in vivo*. Mechanistically, the combination suppresses AKT activity and promotes AKT degradation, ultimately leading to apoptotic cell death. Taken together, these findings support the potential of combined Canagliflozin and Brusatol treatment as an effective therapeutic approach for LKB1-KEAP1 co-mutant NSCLCs.

## Introduction

Lung cancer remains one of the most prevalent cancer types globally and is characterized by a high mortality rate. Approximately 85% of lung cancers are histopathologically classified as non-small cell lung cancer (NSCLC) 1. Based on histological features, the two predominant subtypes of NSCLC are adenocarcinoma (LUAD), which accounts for 40%-50% of cases, and squamous cell carcinoma (LUSC), comprising 20%-30% of cases 2. Despite significant advancements in therapeutic strategies that have improved clinical outcomes, the five-year survival rate for NSCLC patients remains alarmingly low at less than 20% 2, 3.

Liver kinase B1 (LKB1, encoded by *STK11*) is a crucial tumor suppressor that regulates cellular metabolism through activation of its direct downstream effector, AMP-activated protein kinase (AMPK), a central energy sensor 4, 5. LKB1 directly phosphorylates and activates AMPK under metabolic stress. AMPK activation suppresses anabolic metabolism and promotes catabolic processes, including glycolysis and autophagy, to maintain ATP production 6, 7. In NSCLC, LKB1 is the second most frequently altered tumor suppressor, with loss-of-function mutations occurring in 15-30% of cases across both LUAD and LUSC subtypes 8-11. These mutations contribute to tumor progression by disrupting energy homeostasis, redox balance, and anti-tumor immunity 6. Our prior work demonstrated that LKB1-mutant NSCLC cells are highly susceptible to cell death under glucose deprivation. Mechanistically, glucose starvation induces oxidative stress, which causes AMPK oxidation and inactivation in the absence of functional LKB1, ultimately leading to cell death 7.

Canagliflozin is an FDA-approved antihyperglycemic agent for type 2 diabetes that inhibits sodium-glucose co-transporter-2 (SGLT2), thereby blocking renal glucose reabsorption and promoting urinary glucose excretion 12-14. While SGLT2 is predominantly expressed in renal proximal tubules, accumulating studies have reported elevated SGLT2 levels in metastatic lung cancer cells 15. Although studies have highlighted the potential anti-tumour effects of SGLT2 inhibitors 16-18, it remains unclear whether SGLT2 inhibitors such as Canagliflozin display selectivity against cancers with specific genetic profiles. Based on our earlier study that LKB1-mutant NSCLCs are highly sensitive to glucose starvation 7, 19, it was important to determine whether Canagliflozin could selectively kill NSCLC cells harboring LKB1 mutation.

Kelch-like ECH-associated-protein 1 (KEAP1) functions as a central regulator of the antioxidant response pathway by acting as a negative regulator of NRF2, a transcription factor essential for cellular adaptation to oxidative stress 20, 21. Under homeostatic conditions, NRF2 is ubiquitously expressed but maintained at low level through KEAP1-mediated proteasomal degradation 22, 23. Loss-of-function mutations in *KEAP1* occur in approximately 17% of LUAD cases 24. Disruption of KEAP1-Cullin3 ligase activity leads to NRF2 stabilization and nuclear retention, thereby activating the transcription of antioxidant genes. This adaptive response confers resistance to oxidative damage and apoptosis, highlighting the KEAP1-NRF2 axis as a critical survival mechanism in cancer cells 25.

Brusatol, a natural compound isolated from the medicinal plant *Brucea javanica*, has been reported to suppress NRF2 activity by promoting its degradation and disrupting antioxidant and detoxification pathways 26-28. In addition to its effects on NRF2 signalling, Brusatol has shown broader anti-tumour activities in multiple cancer types. For example, in pancreatic cancer, Brusatol has been reported to enhance gemcitabine efficacy by abrogating gemcitabine-induced NRF2 activation and increasing ROS accumulation 28. In renal cell carcinoma, Brusatol suppresses tumour growth through regulation of the PTEN-PI3K-AKT pathway 29. In NSCLC, Brusatol induces ROS-mediated mitochondrial apoptosis and enhances radiosensitivity by increasing ROS production and DNA damage 27. In this study, we systematically evaluated the synergistic anti-tumor efficacy of combined Canagliflozin and Brusatol treatment in LKB1-KEAP1 co-mutant NSCLC models both *in vitro* and *in vivo*. Our findings provide preclinical evidence supporting the development of combined Canagliflozin and Brusatol treatment as a potential therapeutic strategy for NSCLC patients with LKB1-KEAP1 co-mutations.

## Materials and Methods

### Cells and cell culture

A549, H460, H1299, H358, and HEK-293T cells were purchased from American Type Culture Collection (ATCC, USA). Except HEK-293T cells, all cell lines were cultured in Roswell Park Memorial Institute (RPMI) 1640 medium (Gibco, 11875093) supplemented with 10% (v/v) fetal bovine serum (FBS; Gibco, 10099141C) and 1% (v/v) penicillin/streptomycin (Gibco, 15140122). HEK-293T cells were cultured in Dulbecco's Modified Eagle Medium (DMEM; Gibco, 11965092) supplemented with 10% (v/v) FBS (Gibco, 10099141C) and 1% (v/v) penicillin/streptomycin (Gibco, 15140122). All cell line were maintained at 37°C in a humidified incubator with 5% CO_2_. For drug-treatment experiments performed under low-glucose conditions, cells were cultured in RPMI 1640 medium containing 5 mM glucose to mimic physiological blood glucose level. 5 mM glucose RPMI 1640 medium was prepared by dissolving D-(+)-glucose (Sigma, G7021) in glucose-free RPMI 1640 medium (Gibco, 11879020).

### Reagents and antibodies

The following chemicals were purchased from MedChemExpress: Brusatol (HY-19543), Ferrostatin-1 (HY-100579), GSK-872 (HY-101872A), and hydroxychloroquine (HY-B1370). MG132 (S2619) and Bafilomycin A1 (S1413) were purchased from Selleckchem. Z-VAD (V116) and N-Acetyl-L-cysteine (A9165) were purchased from Sigma-Aldrich. Canagliflozin (A11100) was obtained from Adooq Bioscience. The following primary antibodies were used: AKT (9272, 1:1000), caspase-7 (12827, 1:1000), KEAP1 (8047, 1:1000), LKB1 (3050, 1:1000), PARP (9542, 1:1000), phospho-AKT S473 (4060, 1:1000), and phospho-AMPK T172 (2535, 1:1000) from Cell Signalling Technology; AMPK (10929-2-AP, 1:1000) and 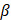-actin (66009-1-Ig, 1:5000) were purchased from Proteintech; GAPDH (ab8245, 1:5000) and NRF2 (ab137550, 1:1000) from Abcam; ubiquitin (sc-8017, 1:1000) from Santa Cruz Biotechnology.

### Cell viability assay

Cell viability was detected using the propidium iodide exclusion assay (Sigma, P4170). Cells were plated in a 12-well plate one day before treatment. After the designed treatments, medium and cells were all collected by trypsin digestion and centrifuged at 300
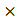
g for 4 minutes at 4°C. The supernatant was discarded, and the pellet was washed with cold PBS twice, then resuspended in the cold PBS containing 5
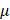
g/ml PI staining reagent and immediately subjected to flow cytometry. Ten thousand cells were analyzed for PI fluorescence intensity using CytoExpert (Beckman Coulter). Data were analyzed using FlowJo_V10 software.

### Immunoblotting

Cells were lysed in SDS lysis buffer containing 20% (v/v) glycerol, 2% (w/v) SDS, 62.5mM Tris-HCl (pH6.8), 2mM DTT, supplemented with 1
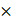
phosphatase and protease inhibitor cocktails. Cell lysates were boiled for 5 minutes, and protein concentration was determined by bicinchoninic acid (BCA) protein assay (Bio-Rad, 5000201). Lysates were mixed with 4
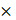
Laemmli loading buffer (Bio-Rad, 1610747). Protein samples (10
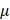
g) were loaded onto SDS-polyacrylamide gels and separated by SDS-PAGE at a constant voltage of 80V, followed by transfer to methanol-activated poly(vinylidene fluoride) (PVDF) membranes at a constant current of 350 mA for 2 hours. Membranes were blocked with SuperBlock T20 (TBS) blocking buffer (Thermo Fisher, ZC386749) for 1 hour at room temperature and then incubated with primary antibodies overnight at 4°C. After primary antibody incubation, membranes were washed three times with Tris-buffered saline containing 0.1% (v/v) TWEEN20 (TBST), with 10 minutes each, and then incubated with the secondary antibodies for 1 hour at room temperature. Chemiluminescence signals were detected using a Bio-Rad ChemiDoc Touch Imaging system. Band intensities were analyzed using Image Lab software.

### ROS measurements

Intracellular ROS levels were measured using CellROX Oxidative Stress Reagent (Thermo Fisher, C10444). Cells were seeded in a 12-well plate. 30 minutes before the end of treatment, CellROX reagent was added to the cells at a final concentration of 5
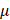
M, followed by incubation at 37°C for 30 minutes. Cells were then washed twice with PBS and harvested by trypsin digestion. Collected cells were centrifugated at 300
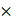
g for 4 minutes at 4°C, and the cell pellet was washed twice with cold PBS. Cells were then resuspended in cold PBS and subjected to flow cytometry (CytoExpert, Beckman Coulter). Data were analyzed using FlowJo_V10 software.

### RNA extraction and real time-PCR (RT-PCR)

Cells were seeded in 12-well plate one day before treatment. After treatment, cells were harvested by trypsin digestion followed by centrifugation at 300
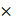
g for 4 minutes. Cell pellet was washed once with PBS and centrifuged again. The pellet was directly subjected to RNA extraction. Total RNA was isolated using the RNAeasy™ Animal RNA Isolation Kit with Spin Column (Beyotime, R0027) according to the manufacturer's instructions. RNA concentration and purity were evaluated by a Nanodrop spectrophotometer. cDNA (1
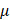
g) was synthesized from the total extracted RNA using the iScript™ cDNA Synthesis Kits (Bio-Rad, 1708891). Real-time PCR was performed using a 10
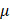
l reaction mixture containing 4
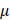
l SsoAdvanced Universal SYBR^®^ Green Supermix (Bio-Rad, 1725274), 1
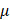
l mixed forward and reverse primers and 200ng cDNA on the Bio-Rad CFX96 Touch Real-Time PCR Detection System. Relative mRNA expression levels were calculated using the 2^-^
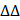
^Ct^ quantification method and normalized to GAPDH. Primer sequences for RT-PCR were as follows:

Human AKT (Forward: 5'-AGCGACGTGGCTATTGTGAAG-3'; Reverse: 5'-GCCATCATTCTTGAGGAGGAAGT-3');

Human GAPDH (Forward: 5'-CCACTCCTCCACCTTTGACG-3'; Reverse: 5'-CCACCACCCTGTTGCTGTAC-3').

### Plasmid construction

To produce retrovirus, pBABE empty vector (Addgene, #1764), pBABE-FLAG-LKB1-WT (Addgene, #8592), and pBABE-FLAG-LKB1-KD (Addgene, #8593) were transfected into Phoenix-AMPHO packaging cells using Lipofectamine^TM^ 3000 Transfection Reagent (Thermo Fisher, L3000015) for 48 hours. Retroviral supernatants were collected, centrifugated at 2000 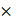
g for 5 minutes, and the supernatant was used to infect A549 and H460 cells in the presence of 5
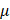
g/ml polybrene for 6 hours.

To produce lentivirus, the KEAP1 construct for overexpression was cloned into the pCDH-CMV vector (Addgene, #72265), and shRNA targeting KEAP1 (Miaoling Biology, P48967) was used for KEAP1 knockdown. HEK-293T cells were seeded in 6-cm dishes at approximately 70% confluency one day before transfection in the DMEM supplemented with 10% (v/v) FBS. Three hours before transfection, the culture medium was replaced with FBS-free DMEM to improve transfection efficacy. PEI MAX-Transfection Grade Linear Polyethyleneimine Hydrochloride (1mg/ml) and lentiviral expression vector were used at a ratio of 3:1, together with 1
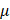
g pCMV-VSV-G and 1
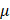
g psPAX2. After 2 hours of transfection, the medium was replaced with DMEM containing 10% (v/v) FBS, and the HEK-293T cells were incubated for an additional 48 hours. Lentiviral supernatants were collected, centrifuged at 2000
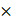
g for 5 minutes, and the supernatant was added to the pre-seeded target cells for further 48 hours of infection. 2
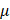
g/ml puromycin (Sigma, P9620) was used to select virally infected cells. Human KEAP1 primer sequences for overexpression were as follows:

Forward: 5'-CTGGAGGATCATACCAAGCAGG-3'.

Reverse: 5'-GGATACCCTCAATGGACACCAC-3'.

### CRISPR-Cas9-mediated gene knockout

Gene knockouts were performed using single-guide RNAs (sgRNAs) and CRISPR-Cas9 technology. sgRNA oligonucleotides targeting human LKB1 were cloned into the pSpCas9(BB)-2A-Puro (PX459) V2.0 vector. The established sgLKB1 plasmid was transfected into H1299 cells using Lipofectamine 3000 reagent. pLenti-Puro-sgAMPK
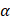
1 (P21236) and pLenti-Puro-sgAMPK
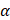
2 (P21235) plasmid were obtained from Miaoling Biology and used to produce lentivirus as described above. Lentiviral particles were added to pre-seeded target cells for 48 hours of infection, and infected cells were selected with 2
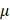
g/ml puromycin for 1 week. Monoclonal cells were then selected and maintained in medium containing 1
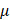
g/ml puromycin. Positive monoclonal cells with LKB1 knockout or AMPK 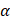
1/ 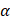
2 double knockout were verified by immunoblotting. The human LKB1 sgRNA sequences were as follows:

Forward: 5'-CACCGTTGCGAAGGACTCCCCAACG-3'.

Reverse: 5'-AAACCGTTGGGGATCCTTCGCAAC-3'.

### Tumour xenograft studies

Male BALB/c nude mice (4-5 weeks old) were obtained from the University of Macau Animal Facility. Mice were randomly divided into four groups and treated with vehicle, Invokana (clinical formulation of Canagliflozin; 200 mg/kg/day), Bursatol (2 mg/kg/day), or the combination of both drugs. Invokana was administered via oral gavage, and Brusatol was delivered intraperitoneally every three days for two weeks. A549 or H1299 (5 × 10^6^ cells in 180
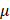
l PBS) cells were subcutaneously implanted into the dorsal flanks of the nude mice. Tumour volume and mouse weight were recorded by callipers and a weighing scale, respectively. Tumour volume was calculated using the formula (a² × b × 0.5), where a and b represent the shortest diameter and longest perpendicular diameters, respectively. At the end of treatments, mice were euthanized by CO₂ asphyxiation. Excised xenograft tissues were weighed, frozen in liquid nitrogen, and stored at -80°C for subsequent analysis.

### Statistical analysis

All experimental procedures were independently repeated at least three times with consistent results. Statistical analyses were conducted using GraphPad Prism software (v7.0), and the corresponding p-values are reported in the figure panels and figure legends. A p-value of < 0.05 was considered statistically significant.

## Results

### Co-occurrence of *STK11* and *KEAP1* mutations in NSCLC

**Figure 1:**
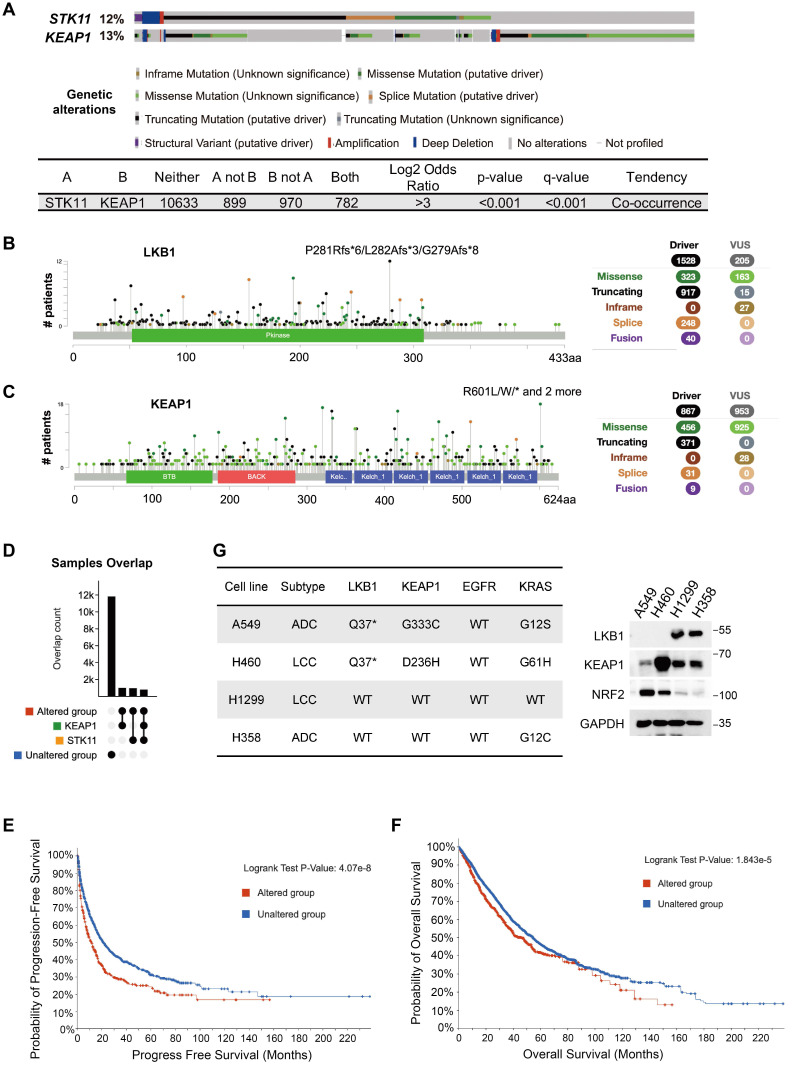
** Co-occurrence of *STK11* and *KEAP1* mutations in NSCLC.** 30 reported NSCLC studies (16 on LUAD, 6 on LUSC, and 8 on both) with a total of 14,700 samples from 12261 patients were pooled for analysis. (A) The mutation spectrum of *STK11* and *KEAP1*. (B and C) Mutation type, frequency, and localization within the protein structure of (B) LKB1 and (C) KEAP1. Circles in distinct colours represent different mutation types. (D) Overlap counts of *STK11* and *KEAP1* mutations in the pooled samples. (E and F) Kaplan-Meier curves showing (E) progression-free survival and (F) overall survival in the entire cohort, comparing samples without mutations in *STK11* or *KEAP1* (unaltered group) with samples harbouring at least one mutation in either gene (altered group). (G) Expression and mutation status of the indicated proteins in four NSCLC cell lines (ADC: adenocarcinoma; LCC: large cell carcinoma).

To investigate the genetic relationship between *STK11* and *KEAP1* mutations, we analyzed genomic data from 30 studies in cBioPortal, comprising 14,700 samples from 12,261 patients. Somatic mutations in *STK11* (12%) and *KEAP1* (13%) were observed at comparable frequencies across the cohorts (Figure 1A). The predominant mutations in *STK11* were truncating (60%), followed by missense (21.2%) and splice site (16.2%) variants (Figure 1B). In contrast, *KEAP1* alterations consisted mainly of missense (52.6%) and truncating (42.5%) mutations (Figure 1C). Strikingly, among the 2,876 samples harbouring at least one alteration in *STK11* or *KEAP1* (termed the altered group), approximately 30% (884 samples) exhibited co-mutations in both genes (Figure 1A and 1D). The prevalence of samples with dual mutations was comparable to that of samples with single-gene mutations, suggesting a pronounced tendency for *STK11* and *KEAP1* co-mutation. Next, we performed survival analyses in patients with *STK11-KEAP1* co-mutations. In the altered group, both progression-free survival (PFS) (p = 4.07e-8 < 0.0001) and overall survival (OS) (p = 1.843e-5 < 0.0001) were significantly reduced compared to the non-altered group (Figure 1E and 1F). Specifically, the median PFS was 11.64 months (95% CI: 9.01-14.66) in the altered group and 21.07 months (95% CI: 18.61-24.99) in the non-altered group, while the median OS was 45.57 months (95% CI: 39.3-51.27) and 54.4 months (95% CI: 50.89-56.71), respectively (Figure 1E and 1F). These results indicated that *STK11*-*KEAP1* co-mutations were associated with a more aggressive disease phenotype, with poorer prognosis. To validate these findings in cancer cell lines, we examined the basal expression levels of LKB1, KEAP1, and NRF2 in four NSCLC cell lines. H1299 and H358 cells retained wildtype LKB1 and KEAP1, whereas A549 and H460 cells were LKB1-deficient, consistent with truncating *STK11* mutations. In addition, although KEAP1 protein expression was preserved in A549 and H460 cells, the G333C and D236H mutations impaired the ability of KEAP1 to bind NRF2 and promote its proteasomal degradation, resulting in constitutive NRF2 activation (Figure 1G).

### Canagliflozin and Brusatol selectively target LKB1-KEAP1 co-mutant NSCLC

**Figure 2:**
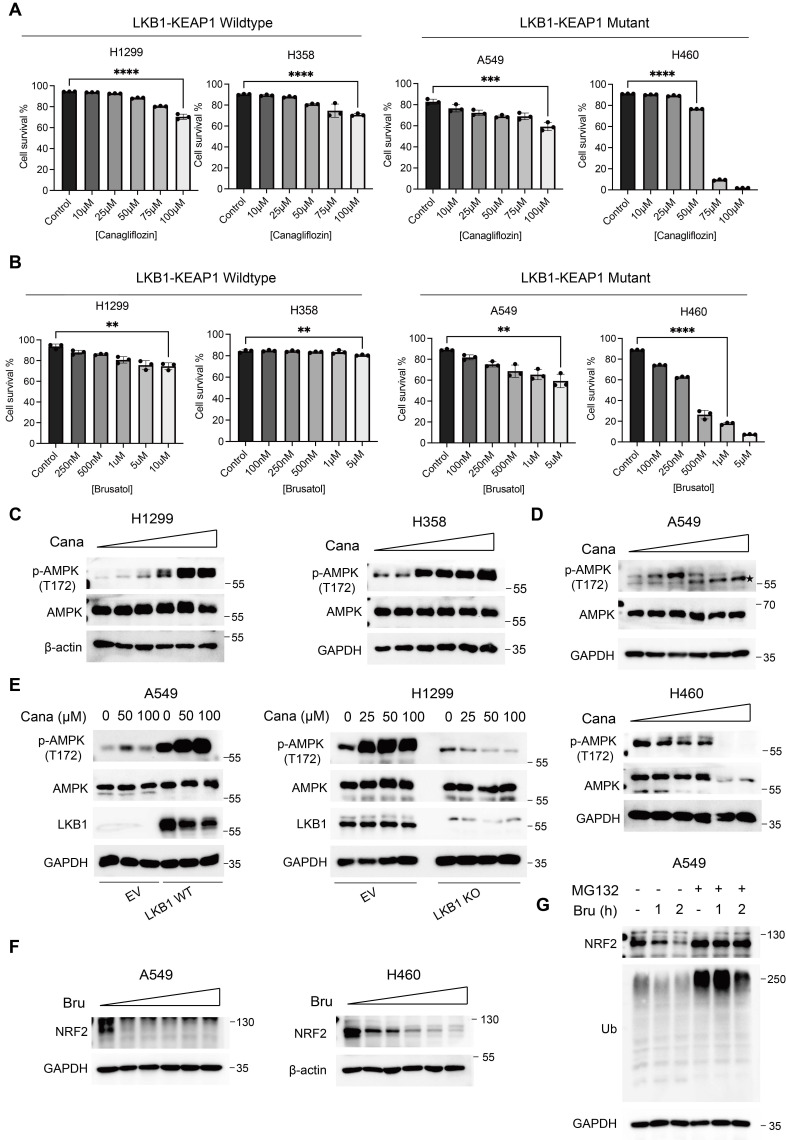
** Canagliflozin and Brusatol selectively target LKB1-KEAP1 co-mutant NSCLC.** (A and B) Cell viability was examined by PI exclusion assay following treatment with (A) Canagliflozin or (B) Brusatol for 48 hours in four NSCLC cell lines. (C and D) Immunoblot results of AMPK activity under Canagliflozin treatment (0, 10, 25, 50, 75, 100 μM) for 12 hours in (C) LKB1-KEAP1 wildtype and (D) LKB1-KEAP1 co-mutant cells. (E) Immunoblot results of AMPK activity under Canagliflozin treatment for 12 hours with different status of LKB1. (F) Immunoblot analysis of NRF2 activity under Brusatol treatment (0.1, 0.25, 0.5, 1, 5 μM) for 6 hours in LKB1-KEAP1 co-mutant cells. (G) Effect of MG132 on Brusatol-treated A549 cells. Cells were pretreated with MG132 (10 μM) for 2 hours, followed by treatment with 500 nM Brusatol treatment for the indicated timepoints. **p < 0.01, ***p < 0.001, ****p < 0.0001 by unpaired t-test. ★ indicates non-specific bands.

To evaluate the potential cytotoxic effects of Canagliflozin and Brusatol, we examined cell survival in a panel of NSCLC cell lines treated with increasing concentrations of each compound. Single treatment with either Canagliflozin or Brusatol induced modest cytotoxicity in LKB1-KEAP1 wildtype cells (H1299 and H358), with cell viability remaining above 80% after 48 hours. In contrast, LKB1-KEAP1 co-mutant cell lines (A549 and H460) exhibited concentration-dependent reductions in survival, with H460 cells showing greater sensitivity than A549 cells at equivalent doses (Figure 2A and 2B). These results indicated that treatment with either Canagliflozin or Brusatol alone selectively killed LKB1-KEAP1 co-mutant NSCLC cells in a dose-dependent manner.

To determine the mechanism underlying the effects of Canagliflozin and Brusatol, we first examined the changes in the LKB1-AMPK pathway. In LKB1 wildtype cells (H1299 and H358), Canagliflozin robustly enhanced AMPK activity (Figure 2C), consistent with its ability to mimic glucose deprivation by blocking glucose uptake. Conversely, LKB1-mutant cells (A549 and H460) displayed reduced AMPK phosphorylation following Canagliflozin treatment (Figure 2D). High doses of Canagliflozin further decreased total AMPK protein levels in H460 cells (Figure 2D), similar to our previous report under glucose starvation 7. Re-expression of wildtype (WT) LKB1, but not empty vector (EV), in A549 cells restored AMPK phosphorylation, whereas LKB1 knockout (KO) in H1299 cells abolished AMPK activation in response to Canagliflozin treatment (Figure 2E). These results thus demonstrated that the high sensitivity of LKB1-mutant cells to Canagliflozin was mediated via AMPK inactivation.

Based on the fact that KEAP1 mutation leads to constitutive NRF2 activation, we next assessed the effects of the NRF2 inhibitor, Brusatol, in KEAP1-mutant NSCLC cells 20. Brusatol reduced NRF2 protein levels at nanomolar concentrations, however, this effect was transient, with NRF2 expression re-appears after prolonged treatment 26. We therefore employed 1
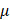
M Brusatol in subsequent experiments (Figure 2F). Pretreating KEAP1-mutant A549 cells with the proteasome inhibitor MG132 reversed the Brusatol-induced NRF2 downregulation (Figure 2G), suggesting that Brusatol promotes NRF2 degradation through ubiquitination and proteasomal degradation mediated by E3 ligases other than KEAP1. The precise mechanism remains to be elucidated in future studies.

### Synergistic effects of Canagliflozin and Brusatol in LKB1-KEAP1 co-mutant NSCLC

**Figure 3:**
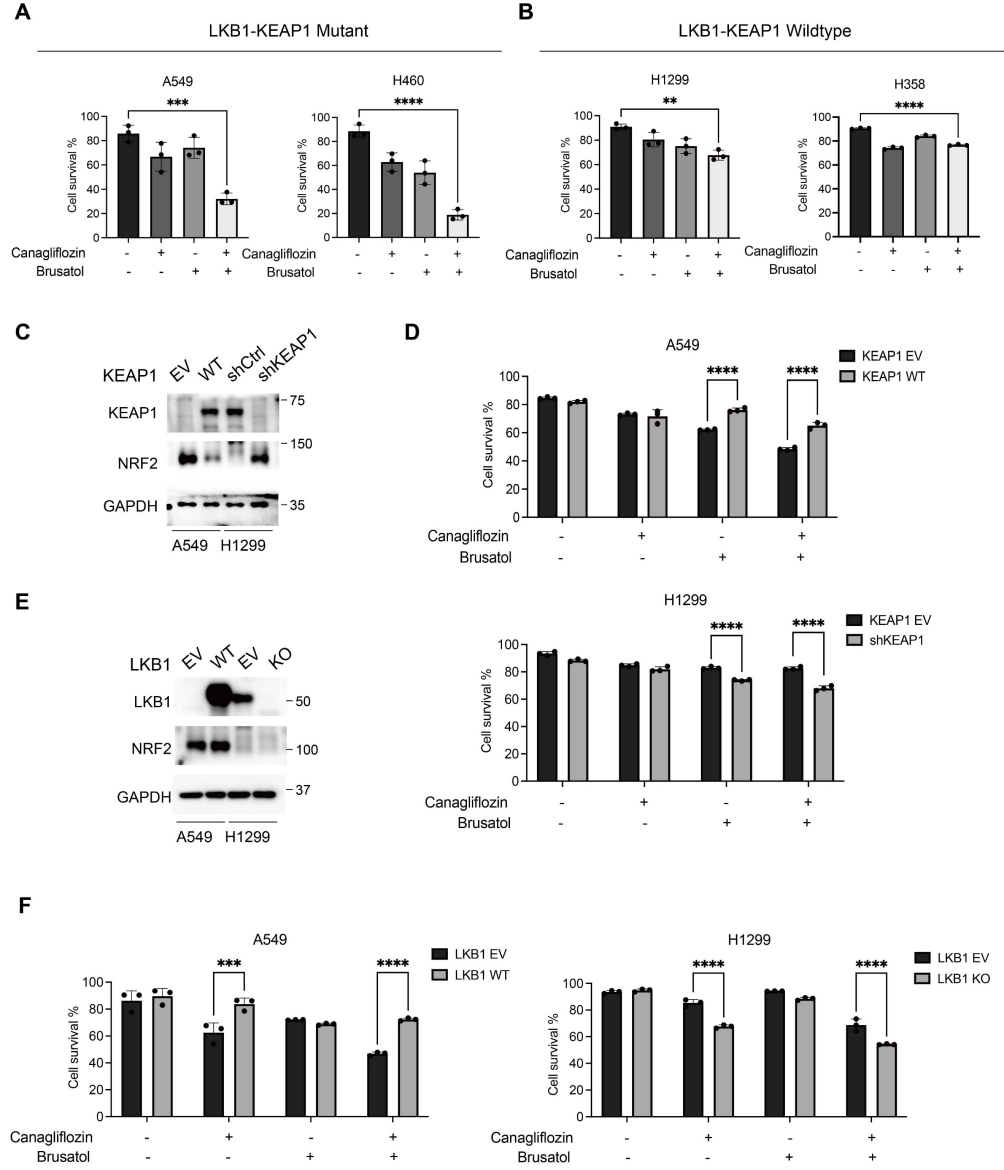
** Synergistic effects of Canagliflozin and Brusatol in LKB1-KEAP1 co-mutant NSCLC.** (A and B) Cell viability was examined by PI exclusion assay following the combination treatment of Canagliflozin (75 μM) and Brusatol (1 μM) for 48 hours in (A) LKB1-KEAP1 co-mutant cells and (B) LKB1-KEAP1 wildtype cells. (C and E) Immunoblot analysis of cells with different status of (C) KEAP1 and (E) LKB1. (D and F) cell viability was measured in cells with different status of (D) KEAP1 and (F) LKB1 treated with Canagliflozin (75 μM or Brusatol (1 μM) for 48 hours. **p < 0.01, ***p < 0.001, ****p < 0.0001 by unpaired t-test (A and B), or two-way ANOVA test (D and F).

To further determine whether combined treatment with Canagliflozin and Brusatol exerts a synergistic effect, we treated A549, H460, H1299, and H358 cells with either single agents or the combination and assessed cell viability by PI exclusion assay. LKB1-KEAP1 co-mutant cell lines (A549 and H460) exhibited significant cell death following combined treatment with Canagliflozin (75
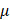
M) and Brusatol (1
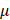
M) (Figure 3A). The synergistic effect of the combination was further confirmed by the Combination Index analysis 30, 31. In contrast, no obvious synergistic effect was observed in LKB1-KEAP1 wildtype cells (H1299 and H358) under the same treatment conditions (Figure 3B). Given the frequent co-occurrence of LKB1 and KEAP1 mutations in NSCLC, we next examined the effects of Canagliflozin and Brusatol in NSCLC cells carrying single mutations in either LKB1 or KEAP1 using genetic manipulation approaches. Restoration of WT KEAP1 in A549 cells reduced NRF2 levels, whereas KEAP1 knockdown (shKEAP1) in H1299 cells elevated NRF2 expression (Figure 3C). Brusatol alone or in combination with Canagliflozin elicited pronounced cytotoxicity in cells with KEAP1 dysregulation than in their wildtype counterparts. Conversely, Canagliflozin alone caused comparable cytotoxic effects regardless of KEAP1 status (Figure 3D). Given that KEAP1 regulates NRF2 expression via ubiquitination and proteasomal degradation, restoration of KEAP1 could be expected to confer cytoprotection, whereas KEAP1-mutant cells exhibited heightened sensitivity to Brusatol-mediated NRF2 inhibition.

To assess whether a reciprocal relationship exists between LKB1 and NRF2, we used opposite approaches to manipulate LKB1 status by reconstituting WT LKB1 in A549 cells and deleting LKB1 in H1299 cells. Neither intervention altered NRF2 expressions, indicating that LKB1 does not directly regulate NRF2 (Figure 3E). Although LKB1-deficient cells were highly sensitive to Canagliflozin alone or in combination with Brusatol, LKB1 status did not affect sensitivity to Brusatol alone (Figure 3F). These findings demonstrated that the efficacy of Brusatol depends on the KEAP1-NRF2 axis but is independent of LKB1, whereas the activity of Canagliflozin depends on LKB1 status rather than KEAP1.

### Combination treatment of Canagliflozin and Brusatol induces apoptosis in LKB1-KEAP1 co-mutant NSCLC

**Figure 4:**
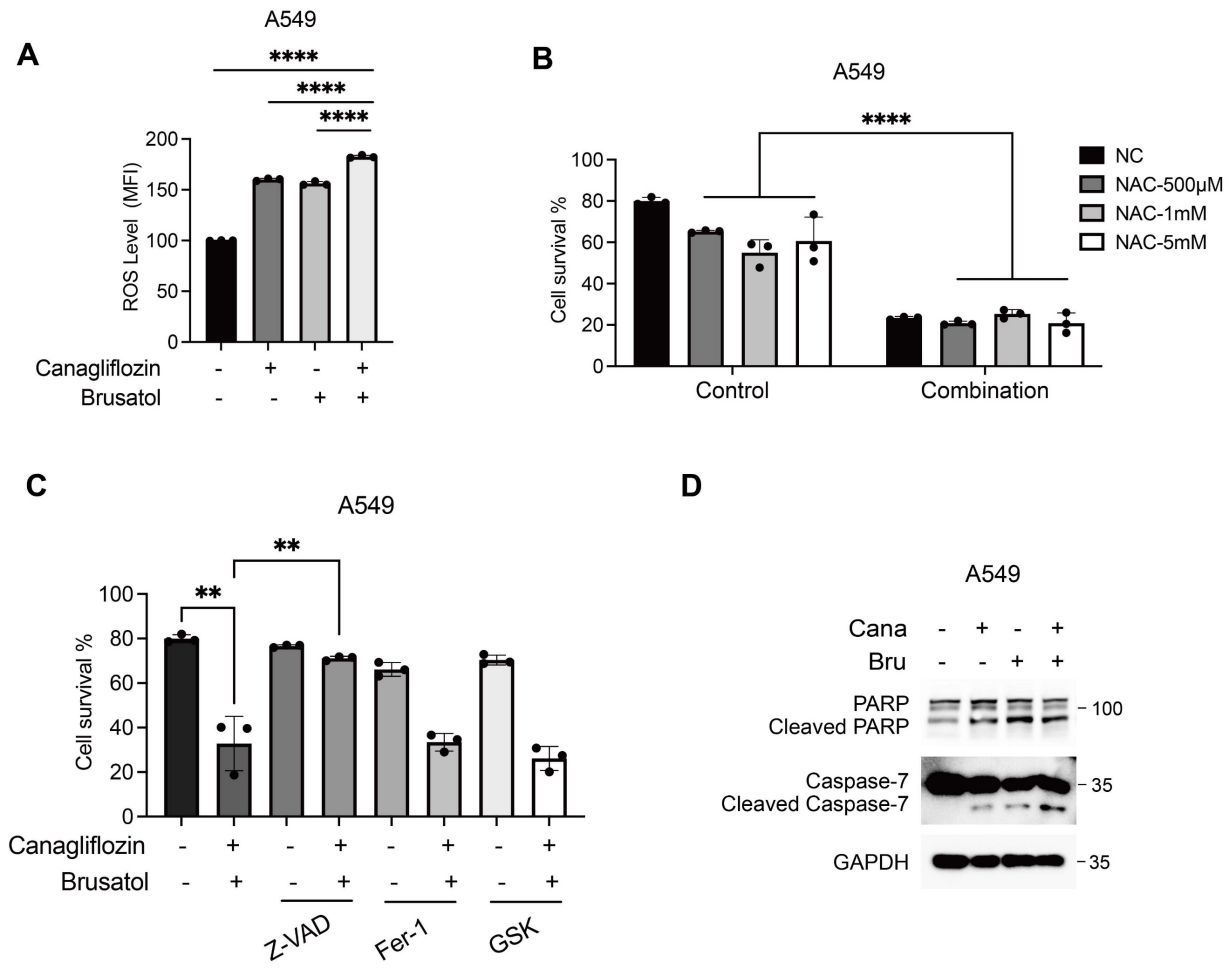
** Combination treatment of Canagliflozin and Brusatol induces apoptosis in LKB1-KEAP1 co-mutant NSCLC.** (A) Intracellular ROS levels in A549 cells following single or combined treatment with Canagliflozin (75 μM) and Brusatol (1 μM) in A549 for 18 hours. (B) Effect of NAC on combination treatment-induced cell death. Cells were pretreated with the indicated concentration of NAC for 2 hours, followed by combination treatment for 48 hours. (C) Effects of various cell death inhibitors on cytotoxicity induced by the combination treatment. Cells were pretreated with Z-VAD (40 μM), Ferrastatin-1 (10 μM), or GSK-872 (10 μM) for 2 hours, followed by combination treatment for 48 hours, and cell viability was quantified. (D) Immunoblot analysis of apoptotic markers under combination treatment for 24 hours. **p < 0.01 and ****p < 0.0001 by unpaired t-test (A and C) or two-way ANOVA test (B).

In KEAP1-mutant cells, constitutive activation of NRF2 drives transcriptional upregulation of antioxidant genes, reducing intracellular ROS levels and protecting cancer cells from oxidative stress 32, 33. Brusatol counteracts this cytoprotective mechanism by promoting NRF2 ubiquitination and proteasomal degradation, thereby exacerbating ROS accumulation 26. Similarly, glucose starvation has been shown to increase ROS levels, sensitizing LKB1-mutant NSCLC cells through AMPK oxidation and inactivation 7. To investigate the molecular mechanism underlying the synergistic cytotoxicity of Canagliflozin and Brusatol in LKB1-KEAP1 co-mutant NSCLC cells, we measured intracellular ROS levels using the CellROX assay, which primarily detects superoxide. Treatment with either Canagliflozin or Brusatol alone increased the ROS levels compared with the untreated control, while the combination treatment produced a stronger effect (Figure 4A). To further evaluate the functional role of ROS in the observed cytotoxicity, cells were pretreated with the ROS scavenger *N*-acetylcysteine (NAC). Unexpectedly, despite testing multiple concentrations, NAC failed to rescue cell death induced by the combination treatment, suggesting the synergistic effect of these two agents operates via another mechanism (Figure 4B). To delineate the type of cell death induced by co-treatment, we employed pharmacological inhibitors targeting distinct cell death pathways, including the pan-caspase inhibitor Z-VAD, the ferroptosis inhibitor ferrostatin-1, and the necroptosis inhibitor GSK-872. Pretreatment with Z-VAD, but not ferrostatin-1 or GSK-872, significantly attenuated cell death induced by the combined treatment, indicating that apoptosis is the predominant mechanism (Figure 4C). This conclusion was further supported by increased levels of cleaved PARP and cleaved caspase 7, two apoptotic hallmarks, in cells treated with both Canagliflozin and Brusatol (Figure 4D). Taken together, these results suggest that the synergistic cytotoxicity of Canagliflozin and Brusatol in LKB1-KEAP1 co-mutant NSCLC cells is mediated predominantly by apoptosis rather than by ROS accumulation alone.

### Apoptosis induced by Canagliflozin and Brusatol is mediated by suppression of AKT activity in LKB1-KEAP1 co-mutant NSCLC

**Figure 5:**
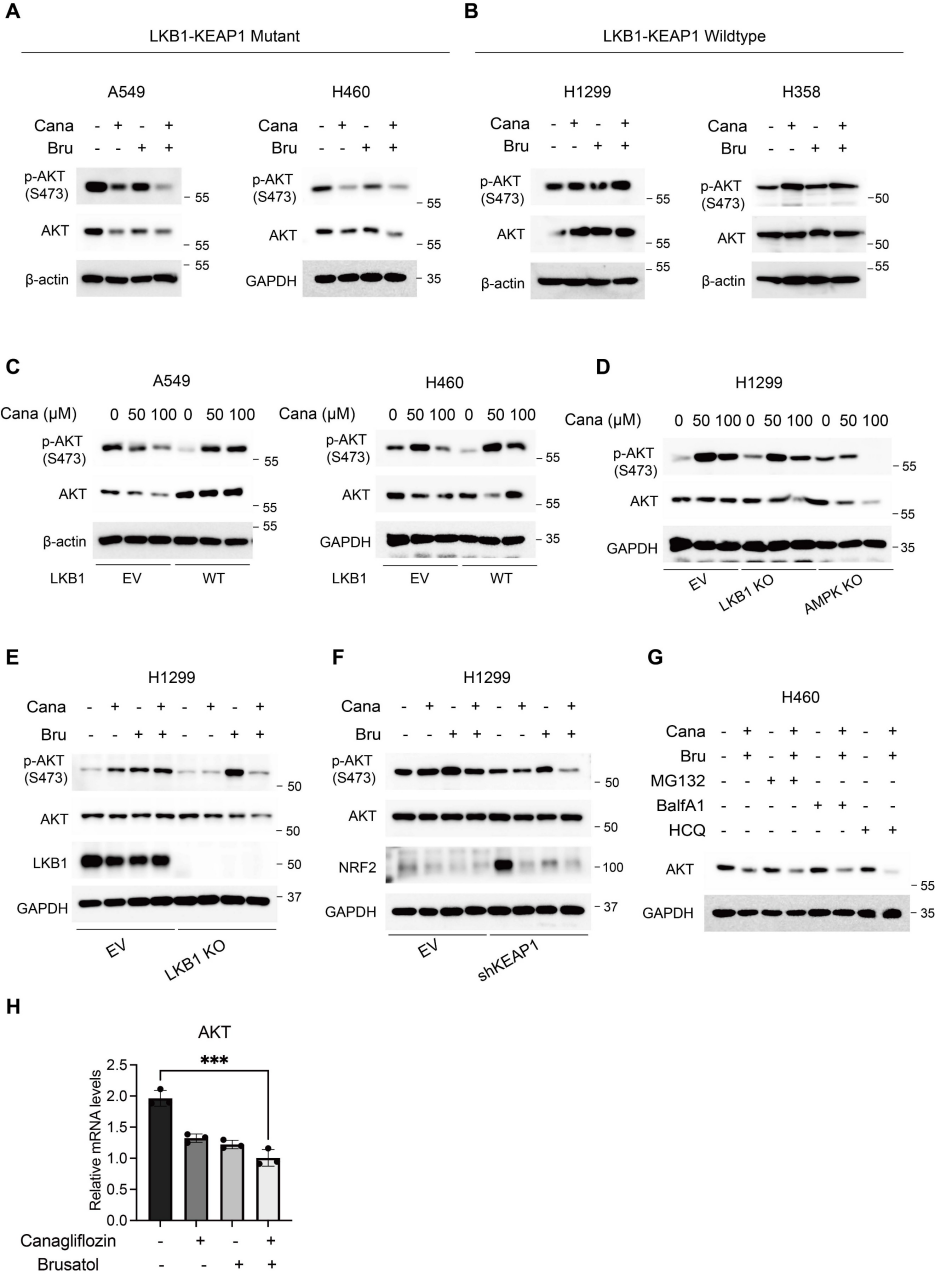
** Apoptosis induced by Canagliflozin and Brusatol is mediated by suppression of AKT activity in LKB1-KEAP1 co-mutant NSCLC.** (A and B) Immunoblot analysis of AKT activity under the combination treatment for 12 hours in (A) LKB1-KEAP1 co-mutant cells and (B) LKB1-KEAP1 wildtype cells. (C and D) Immunoblot results of AKT activity under Canagliflozin treatment for 12 hours with different status of (C) LKB1 and (D) AMPK. (E and F) Immunoblot results of AKT activity in H1299 cells with different status of (E) LKB1 or (F) KEAP1. (G) Effects of various protein degradation pathway inhibitors on AKT downregulation induced by the combination treatment. Cells were pretreated with MG132 (10 μM), Bafilomycin A1 (BalfA1, 100 μM), or hydroxychloroquine (HCQ, 20 mM) for 2 hours, followed by combination treatment for 12 hours. (H) RT-PCR analysis of AKT mRNA levels in H460 cells upon single-agent or combined treatments for 12 hours. ***p < 0.001 by unpaired t-test.

Both Canagliflozin and Brusatol have been implicated in modulating the PI3K/AKT/mTOR signalling axis to influence tumorigenesis 29, 34. Brusatol, for instance, suppressed renal cancer cell proliferation by upregulating PTEN, a key tumour suppressor with lipid and protein phosphatase activities, thereby inhibiting the PI3K-AKT pathway 29. In LKB1-KEAP1 co-mutant cells, treatment with either Canagliflozin or Brusatol alone significantly reduced both phosphorylated AKT and total AKT levels, whereas the combined treatment further suppressed AKT activity (Figure 5A). In contrast, LKB1-KEAP1 wildtype cells showed minimal changes in phosphorylated AKT and total AKT levels following the co-treatment (Figure 5B). Given the established role of AKT as a master regulator of cell survival and anti-apoptotic signalling 35, 36, its inhibition in LKB1-KEAP1 co-mutant cells following treatment with Canagliflozin and Brusatol suggests that the suppression of this anti-apoptotic pathway is a primary driver of their high susceptibility to the combination treatment.

Next, we further elaborated on the respective roles of LKB1 and KEAP1 in regulating AKT activity. Restoration of functional LKB1 in A549 and H460 cells, both of which harbour LKB1-KEAP1 co-mutations, reversed the Canagliflozin-induced suppression of AKT activity, as evidenced by recovery of both phosphorylated and total AKT levels (Figure 5C). The reduction in phosphorylated AKT was consistent with the decline in phosphorylated AMPK observed following Canagliflozin treatment (Figure 2E). Similarly, genetic deletion of LKB1 or its downstream effector AMPK in H1299 cells attenuated AKT activation in response to Canagliflozin (Figure 5D), highlighting the requirement for an intact LKB1-AMPK axis in maintaining AKT activity. Furthermore, combined treatment with Canagliflozin and Brusatol strongly suppresses AKT activity in H1299 cells depleted of either LKB1 or KEAP1 (Figure 5E and 5F). Collectively, these findings demonstrated that both LKB1 and KEAP1 are required to maintain AKT activity.

Finally, we sought to clarify the mechanism of AKT degradation during combination treatment. It is interesting to note that neither proteasome inhibitor MG132 nor the lysosomal inhibitors bafilomycin A1 and hydroxychloroquine rescued the reduction in AKT protein levels induced by the co-treatment of Canagliflozin and Brusatol, indicating that AKT downregulation is independent of the canonical ubiquitin-proteasomal or lysosomal degradation systems (Figure 5G). To test the possibility that AKT reduction might instead occur at the transcriptional level, we measured AKT mRNA levels. As expected, single treatment with either Canagliflozin or Brusatol largely reduced AKT mRNA levels, and combination treatment further exacerbated this effect (Figure 5H). Together, these data indicated that the reduction in total AKT protein observed during combination treatment was driven by transcriptional downregulation of AKT. Future studies are needed to define the precise mechanisms controlling AKT transcription under this setting.

### Combination treatment of Canagliflozin and Brusatol exerts anti-tumour effects in the xenograft model *in vivo*

**Figure 6:**
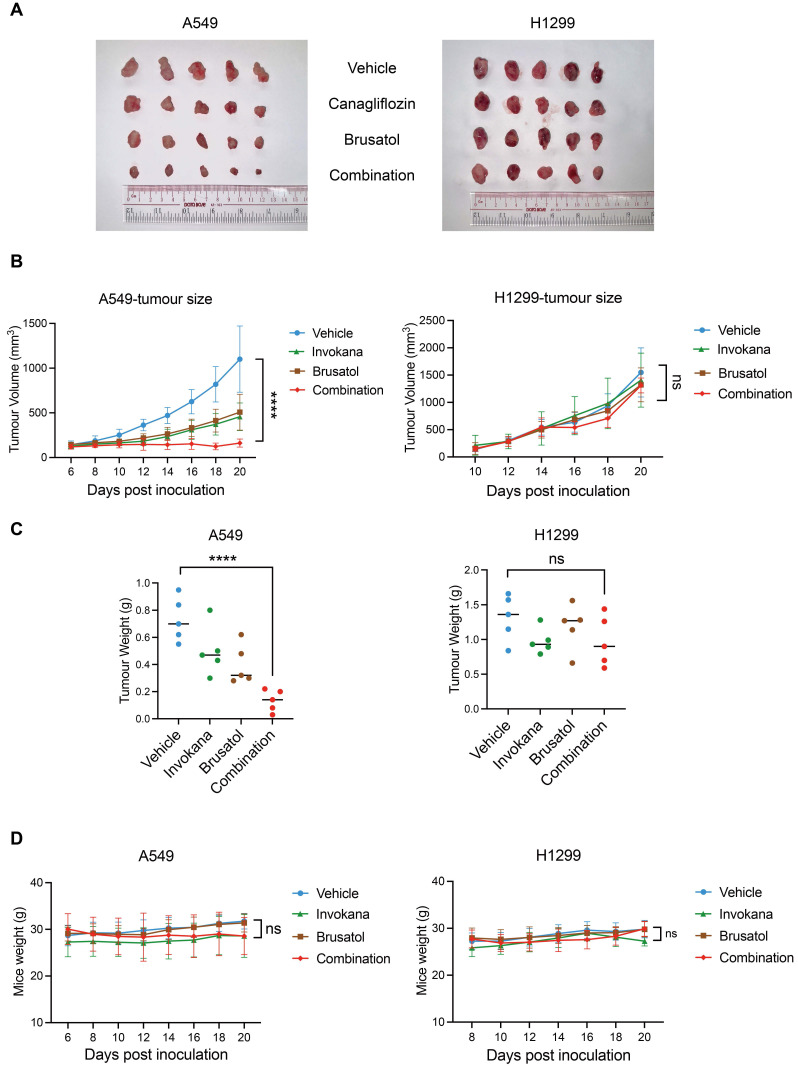
** Combination treatment of Canagliflozin and Brusatol exerts anti-tumour effects in the xenograft model *in vivo.*** (A) Images of A549- and H1299-derived xenograft tumours at the endpoint of treatment with vehicle, single-agent treatment, or combined treatments with Invokana (200 mg/kg/day) and Brusatol (2 mg/kg/day). (B) Tumour volume measurements over the course of the treatment in A549 and H1299 xenografts (A549: n=5; H1299: n=5). (C) Final tumour weights of A549- and H1299-derived xenografts under the treatment conditions shown in (A). (D) Body weight measurements of mice during the treatment period. ****p < 0.0001 by two-way ANOVA test (B and D), or unpaired t-test (C). Quantitative data are represented as mean ± SD (B and D) or medium (C).

To evaluate the anti-tumour efficacy of Canagliflozin and Brusatol co-treatment *in vivo*, we established xenograft models using male BALB/c nude mice implanted with A549 (LKB1-KEAP1co-mutant) or H1299 (LKB1-KEAP1 wildtype) cells. Mice were randomly and unbiasedly allocated into four groups: vehicle, Invokana (clinical formulation of Canagliflozin; 200 mg/kg/day), Brusatol (2 mg/kg/day), or the combination treatment. In A549 xenografts, treatment with either Invokana or Brusatol alone modestly suppressed tumour growth, whereas the combination treatment induced tumour regression, as evidenced by a substantial reduction in tumour volume (Figure 6A and 6B). In contrast, neither single-treatment nor the combination treatment reduced tumour growth in H1299 xenografts (Figure 6A and 6B). Final tumour weights were consistent with the tumour volume (Figure 6C). Notably, no significant loss of body weight was observed in any treatment group (Figure 6D), indicating minimal systemic toxicity. These results thus demonstrated that co-treatment with Invokana and Brusatol exerts selective therapeutic efficacy in LKB1-KEAP1 co-mutant NSCLC *in vivo.*

## Discussion

**Figure 7:**
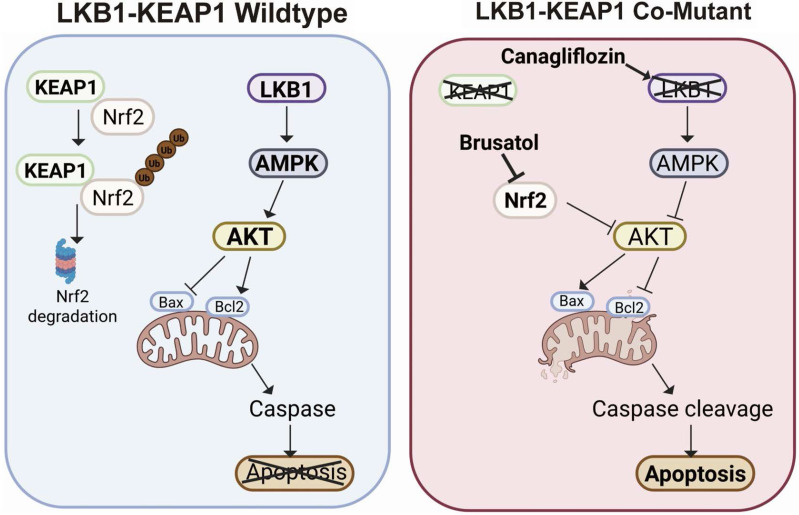
** Schematic model of Canagliflozin and Brusatol combination treatment selectively mediate anti-tumour effects in LKB1-KEAP1 co-mutant NSCLC.** In normal LKB1-KEAP1 wildtype cells, NRF2 is constitutively degraded by KEAP1 via proteosomal degradation. Under stress conditions, intact AKT signalling prevents apoptosis in the presence of functional LKB1 and AMPK. In LKB1-KEAP1 co-mutant NSCLC cells, Canagliflozin and Brusatol individually suppress AMPK activity and promote NRF2 degradation, respectively. Combined treatment with both drugs synergistically induces cell apoptosis through inhibition of AKT activity.

LKB1 (encoded by *STK11*) and KEAP1 are two vital tumour suppressors in NSCLC. Patients with NSCLC harbouring LKB1 or KEAP1 mutations are often less responsive to current targeted therapies, including EGFR inhibitors 37, 38. Therefore, the development of new therapeutic strategies for NSCLC patients with these genetic alterations remain an important unmet need. In this study, we used four well-characterized NSCLC cell lines with distinct LKB1 and KEAP1 genotypes to model an aggressive molecular subset of human NSCLC. We first confirmed the co-occurrence of LKB1 and KEAP1 mutations in NSCLC and then evaluated two pharmacological agents, Canagliflozin, a selective SGLT2 inhibitor, and Brusatol, an NRF2 inhibitor, as a therapeutic strategy for LKB1-KEAP1 co-mutant NSCLC. Our data showed that combined treatment with Canagliflozin and Brusatol selectively killed NSCLC cells harbouring LKB1-KEAP1 co-mutations, both *in vitro* and *in vivo*, by suppressing AKT activity and ultimately inducing apoptosis (as summarized in Figure 7). Taken together, our findings identify a potential therapeutic strategy for this particularly aggressive molecular subtype and may lay the foundation for future translational clinical studies.

Canagliflozin, a selective SGLT2 inhibitor, was approved by the FDA in 2013 as an oral antihyperglycemic agent for the treatment of type 2 diabetes mellitus (T2DM). Recently, accumulating evidence has suggested that Canagliflozin exerts biological effects beyond glycaemic control, including cardiovascular and renal protection 12. Notably, unlike the other SGLT2 inhibitors Dapagliflozin and Empagliflozin, Canagliflozin has been reported to inhibit mitochondrial electron transport chain (ETC) complex I, thereby reducing oxidative phosphorylation and ATP production and potentially increasing mitochondrial ROS levels 39. Given that high SGLT2 expression has been reported in both lung premalignant cells and metastatic lung cancer lesions, targeting SGLT2 with Canagliflozin may represent a potential therapeutic strategy in NSCLC 19. In line with this possibility, our data show that Canagliflozin selectively kills LKB1-mutant NSCLC cells. Mechanistically, in the absence of LKB1, Canagliflozin mimics the effect of glucose starvation, resulting in the inactivation of AMPK and AKT, and ultimately leading to apoptotic cell death. These findings support the notion of repurposing Canagliflozin as a cancer therapeutic agent for NSCLC with LKB1 mutations.

As another key tumour suppressor in NSCLC, KEAP1 negatively regulates NRF2 through the Cullin 3-based E3 ubiquitin ligase complex. Mutation of KEAP1 disrupts this regulatory mechanism, leading to stabilization and aberrant activation of NRF2, a major transcriptional factor involved in antioxidant defence and cell survival 40. It is noteworthy that NSCLC cells harbouring KEAP1 mutations often become highly dependent on NRF2 signalling for tumour maintenance, a phenomenon termed 'NRF2 addiction' 41. This concept provides the rationale for testing Brusatol as a therapeutic agent in KEAP1-mutant NSCLC. Previous studies have demonstrated that Brusatol exerts pro-apoptotic activity in multiple cancer models, including NSCLC and pancreatic adenocarcinoma 27, 28. However, the anti-cancer function of Brusatol has not been clearly linked to specific genetic backgrounds. In this study, we found that Brusatol selectively killed NSCLC cells with KEAP1 mutation, further supporting the therapeutic potential of genotype-based treatment in this context.

A hallmark metabolic feature of cancer cells is the Warburg effect, which refers to the preferential reliance on aerobic glycolysis 42-44. Recent studies have attempted to develop targeted therapeutic strategies for LKB1-KEAP1 co-mutant tumours on the basis of their metabolic vulnerabilities. It has been reported that concurrent loss of LKB1 and KEAP1 promotes metabolic reprogramming from glycolytic dependence towards glutaminolysis 45, 46. In addition, targeting glutaminase, which converts glutamine to glutamate, has been shown to sensitize LKB1-KEAP1 co-mutant cells to radiotherapy 37. Thus, our study provides a new line of evidence that combined treatment with Canagliflozin and Brusatol may represent an effective strategy for NSCLC with LKB1-KEAP1 co-mutations.

In summary, our study proposes a novel targeted therapeutic strategy for NSCLC harbouring LKB1-KEAP1 co-mutations through the combined use of Canagliflozin and Brusatol. This new therapeutic approach may represent a potential treatment option for patients with this aggressive molecular subtype. Nevertheless, several limitations of this study should be noted. Although we showed that combined Canagliflozin and Brusatol treatment exerted anti-tumour effects in NSCLC cell lines and xenograft models, the study was based on a limited number of preclinical systems. Further validation in the patient-derived xenografts (PDXs) will be important, as this model better reflects the complex heterogeneity of human tumours. In addition, the upstream mechanism linking LKB1-KEAP1 co-mutation to AKT regulation was not fully elucidated. In particular, although AKT mRNA levels were reduced, the transcriptional regulators responsible for this effect were not investigated in the present study. Therefore, future studies will be needed to clarify this mechanism, which will be important for strengthening the translational potential of this therapeutic approach.
